# Development and characterization of anti‐glycopeptide monoclonal antibodies against human podoplanin, using glycan‐deficient cell lines generated by CRISPR/Cas9 and TALEN

**DOI:** 10.1002/cam4.954

**Published:** 2017-01-19

**Authors:** Mika K. Kaneko, Takuro Nakamura, Ryusuke Honma, Satoshi Ogasawara, Yuki Fujii, Shinji Abe, Michiaki Takagi, Hiroyuki Harada, Hiroyoshi Suzuki, Yasuhiko Nishioka, Yukinari Kato

**Affiliations:** ^1^ Department of Regional InnovationTohoku University Graduate School of Medicine2‐1 Seiryo‐machi, Aoba‐kuSendaiMiyagi980‐8575Japan; ^2^ Department of Orthopaedic SurgeryYamagata University Faculty of Medicine2‐2‐2 Iida‐nishiYamagata990‐9585Japan; ^3^ Department of Clinical Pharmacy Practice PedagogyGraduate School of Biomedical SciencesTokushima University1‐78‐1 Sho‐machiTokushima770‐8505Japan; ^4^ Department of Respiratory Medicine and RheumatologyGraduate School of Biomedical SciencesTokushima University3‐18‐15 Kuramoto‐choTokushima770‐8503Japan; ^5^ Oral and Maxillofacial SurgeryGraduate School of Medical and Dental SciencesTokyo Medical and Dental University1‐5‐45, YushimaBunkyo‐kuTokyo113‐8510Japan; ^6^ Department of Pathology and Laboratory MedicineSendai Medical Center2‐8‐8, Miyagino, Miyagino‐kuSendaiMiyagi983‐0045Japan

**Keywords:** Epitope, glycopeptide, monoclonal antibody, podoplanin, sialic acid

## Abstract

Human podoplanin (hPDPN), which binds to C‐type lectin‐like receptor‐2 (CLEC‐2), is involved in platelet aggregation and cancer metastasis. The expression of hPDPN in cancer cells or cancer‐associated fibroblasts indicates poor prognosis. Human lymphatic endothelial cells, lung‐type I alveolar cells, and renal glomerular epithelial cells express hPDPN. Although numerous monoclonal antibodies (mAbs) against hPDPN are available, they recognize peptide epitopes of hPDPN. Here, we generated a novel anti‐hPDPN mAb, LpMab‐21. To characterize the hPDPN epitope recognized by the LpMab‐21, we established glycan‐deficient CHO‐S and HEK‐293T cell lines, using the CRISPR/Cas9 or TALEN. Flow cytometric analysis revealed that the minimum hPDPN epitope, in which sialic acid is linked to Thr76, recognized by LpMab‐21 is Thr76–Arg79. LpMab‐21 detected hPDPN expression in glioblastoma, oral squamous carcinoma, and seminoma cells as well as in normal lymphatic endothelial cells. However, LpMab‐21 did not react with renal glomerular epithelial cells or lung type I alveolar cells, indicating that sialylation of hPDPN Thr76 is cell‐type‐specific. LpMab‐21 combined with other anti‐hPDPN antibodies that recognize different epitopes may therefore be useful for determining the physiological function of sialylated hPDPN.

## Introduction

Human podoplanin (hPDPN) is expressed in many cancers, including malignant gliomas, lung cancers, esophageal cancers, malignant mesotheliomas, testicular cancers, bladder cancers, and osteosarcomas 1, 2, 3, 4, 5, 6, 7, 8, 9, 10, 11, 12, 13, and the expression of hPDPN in cancer‐associated fibroblasts contributes to poor prognosis 14, 15, 16, 17, 18, 19. Human PDPN (known as the platelet aggregation‐inducing factor Aggrus) is involved in cancer metastasis 11, 20, 21. We identified C‐type lectin‐like receptor‐2 (CLEC‐2) as an endogenous receptor of hPDPN 22, 23. Moreover, our comparative crystallographic studies of the hPDPN–CLEC‐2 complex 24 revealed that CLEC‐2 binds to hPDPN through residues Glu47 and Asp48 within its platelet aggregation‐stimulating (PLAG) domain as well as to the α2‐6‐linked sialic acid linked to Thr52.

Highly sensitive and specific anti‐hPDPN mAb are required to clarify the physiological function of hPDPN in normal tissues and cancers. Although many anti‐hPDPN mAbs are available, most react with the PLAG1‐PLAG3 of hPDPN 6, 25, 26, 27, 28, 29. We previously established the original technology to produce anti‐glycopeptide mAbs (GpMabs) against hPDPN 30, 31, 32, 33, 34, 35, 36, 37, 38, 39. Here, we generated a novel anti‐hPDPN mAb designated LpMab‐21 that recognizes a sialylated glycopeptide epitope.

Furthermore, to characterize the hPDPN epitope recognized by the LpMab‐21, we need glycan‐deficient CHO‐S or HEK‐293T cell lines. We report the establishment of glycan‐deficient cell lines using the CRISPR/Cas9 or TALEN.

## Materials and Methods

### Cell lines, mice, and human tissues

As described in detail previously 36, 39, the cell lines LN229, HEK‐293T, NCI‐H226, U‐2 OS, Met‐5A, Chinese hamster ovary (CHO)‐K1, and P3U1 were obtained from the American Type Culture Collection (ATCC, Manassas, VA). The HSC‐2 and HSC‐4 cell lines were obtained from the Japanese Collection of Research Bioresources (JCRB) Cell Bank (Osaka, Japan). The MG‐63 cell line was obtained from the Cell Resource Center for Biomedical Research Institute of Development, Aging and Cancer Tohoku University (Miyagi, Japan). The LN319 cell line was provided by Prof. Kazuhiko Mishima (Saitama Medical University, Saitama, Japan) 40. Human lymphatic endothelial cells (LECs), CHO‐S cells, and PC‐10 cells were purchased from Cambrex Corp., East Rutherford, NJ, Thermo Fisher Scientific Inc., (Waltham, MA), and Immuno‐Biological Laboratories Co., Ltd. (Gunma, Japan), respectively. LN229 and CHO‐K1 cells were transfected with the hPDPN plasmids using Lipofectamine 2000 (Thermo Fisher Scientific Inc.) according to the manufacturer's instructions 30.

CHO‐K1, CHO‐K1/hPDPN, CHO‐S, NCI‐H226, PC‐10, and P3U1 cells were cultured in RPMI 1640 medium containing l‐glutamine (Nacalai Tesque, Inc., Kyoto, Japan). LN229, LN229/hPDPN, LN319, HSC‐2, HSC‐4, and HEK‐293T cells were cultured at 37°C in a humidified atmosphere containing 5% CO_2_ in Dulbecco's Modified Eagle's Medium (DMEM) medium containing L‐glutamine (Nacalai Tesque, Inc.) and 10% heat‐inactivated fetal bovine serum (FBS) (Thermo Fisher Scientific Inc.). LECs were cultured in endothelial cell medium EGM‐2MV supplemented with 5% FBS (Cambrex Corp. East Rutherford, NJ). All media contained 100 units/mL of penicillin, 100 *μ*g/mL of streptomycin, and 25 *μ*g/mL of amphotericin B (Nacalai Tesque, Inc.).

Three female BALB/c mice (4‐week‐old) were purchased from CLEA Japan (Tokyo, Japan) and were housed under pathogen‐free conditions. The Animal Care and Use Committee of Tohoku University approved the animal experiments described herein.

The Tokyo Medical and Dental University Institutional Review Board and the Sendai Medical Center Review Board reviewed and approved the use of human cancer tissues. Written informed consent was obtained for using the human cancer tissue samples. Microarrays of normal human tissues were purchased from Cybrdi, Inc. (Frederick, MD).

### Production of glycan‐deficient or PDPN‐knock out cell lines

The HEK‐293T/hPDPN‐knockout (KO) cell line (PDIS‐2) and the LN319/hPDPN‐KO cell line (PDIS‐6) were generated by transfection using CRISPR/Cas9 plasmids (Target ID: HS0000333287) that target PDPN (Sigma‐Aldrich, St. Louis, MO). Plasmids were transfected, using a Gene Pulser Xcell electroporation system (Bio‐Rad Laboratories Inc., Berkeley, CA) 31, 34. PDIS‐2 and PDIS‐6 cells were screened using the NZ‐1 mAb 5. The cell lines CHO‐S/GnT‐1‐KO (PDIS‐9) and CHO‐S/SLC35A1‐KO (PDIS‐14) were generated by transfecting TALEN or CRISPR/Cas9 plasmids, which target hsMgat1 (Wako Pure Chemical Industries Ltd., Osaka, Japan) and SLC35A1 (Target ID: HS0000168432; Sigma‐Aldrich), respectively, using a Gene Pulser Xcell electroporation system. The cell lines HEK‐293T/GnT‐1‐KO (PDIS‐1 or PDIS‐12) and HEK‐293T/SLC35A1‐KO (PDIS‐22) were generated by transfecting TALEN or CRISPR/Cas9 plasmids, which target hsMgat1 (Wako Pure Chemical Industries Ltd., Osaka, Japan) and SLC35A1 (Target ID: HS0000168432; Sigma‐Aldrich), respectively, using a Gene Pulser Xcell electroporation system. PDIS‐1, PDIS‐9, and PDIS‐12 cells were screened using leukoagglutinin from *Phaseolus vulgaris* (L‐PHA). PDIS‐14 and PDIS‐22 cells were screened using *Maackia amrensis* Lectin II (MAL‐II). PDIS‐9 and PDIS‐14 cells were transfected with the human PDPN plasmids using Lipofectamine LTX (Thermo Fisher Scientific Inc.) according to the manufacturer's instructions. Glycan‐deficient cell lines were cultured in RPMI 1640 medium.

### Generation of deletion mutants

Amplified hPDPN cDNA was subcloned into a pCAG‐Ble(Zeo) vector (Wako Pure Chemical Industries Ltd.) with a MAP‐tag, detected by PMab‐1 41, 42, which was added to the N‐terminus using the In‐Fusion HD Cloning Kit (Clontech, Palo Alto, CA). Deletion mutants of hPDPN were generated using the primers as follows:

Sense primers and designation of the corresponding mutant

5′‐AGAAGACAAAAAGCTTGCCAGCACAGGCCAGCC, dN23

5′‐AGAAGACAAAAAGCTTGAAGGCGGCGTTGCCAT, dN37

5′‐AGAAGACAAAAAGCTTGCCGAAGATGATGTGGTG, dN46

5′‐AGAAGACAAAAAGCTTACCAGCGAAGACCGCTA, dN55

5′‐AGAAGACAAAAAGCTTACAACTCTGGTGGCAACA, dN64

5′‐AGAAGACAAAAAGCTTGTAACAGGCATTCGCATC, dN75

5′‐AGAAGACAAAAAGCTTACTTCAGAAAGCACAGTCC, N85

5′‐AGAAGACAAAAAGCTTCAAAGTCCAAGCGCCAC, dN95

5′‐AGAAGACAAAAAGCTTGCCACCAGTCACTCCAC, dN105

Antisense primer

5′‐TCTAGAGTCGCGGCCGCTTACTTGTCGTCATCGT

CHO‐K1 cells were transfected with these plasmids using Lipofectamine LTX (Thermo Fisher Scientific Inc.). Deletion mutants were cultured in RPMI 1640 medium containing l‐glutamine (Nacalai Tesque, Inc.) and 10% heat‐inactivated FBS at 37°C in a humidified atmosphere containing 5% CO_2_. Stable transfectants of CHO‐K1/ssMAP‐hPDPNdN mutants were selected by culturing them in medium containing 0.5 mg/mL Zeocin (InvivoGen, San Diego, CA).

### Production of point mutants

The amplified hPDPN cDNA was subcloned into a pcDNA3 vector (Thermo Fisher Scientific Inc.), and a FLAG epitope tag was added to the C‐terminus. Substitutions of amino acid residues to Ala or Gly in the hPDPN sequence were performed, using a QuikChange Lightning site‐directed mutagenesis kit (Agilent Technologies Inc., Santa Clara, CA) using oligonucleotides containing the desired mutations. CHO‐S or CHO‐K1 cells were transfected with the plasmids using a Gene Pulser Xcell electroporation system (Bio‐Rad Laboratories Inc.). Point mutants were cultured in RPMI 1640 medium containing l‐glutamine.

### Hybridoma production

Three 4‐week‐old female BALB/c mice were immunized by intraperitoneal (i.p.) injection of 1 × 10^8^ LN229/hPDPN cells together with Imject Alum (Thermo Fisher Scientific Inc.) 30. A booster injection was administered i.p. 2 days before the mice were euthanized by cervical dislocation. Spleen cells were harvested and fused with P3U1 cells using PEG1500 (Roche Diagnostics, Indianapolis, IN). The hybridomas were cultured in RPMI 1640 medium containing hypoxanthine, aminopterin, and thymidine selection medium supplement (Thermo Fisher Scientific Inc.). The culture supernatants were screened, using an enzyme‐linked immunosorbent assay (ELISA) and recombinant human PDPN purified from LN229/hPDPN cells 30. Proteins (1 *μ*g/mL) were immobilized on Nunc Maxisorp 96‐well immunoplates (Thermo Fisher Scientific Inc.) for 30 min. After blocking with 1% bovine serum albumin (BSA) in 0.05% Tween20/phosphate‐buffered saline (PBS) (Nacalai Tesque, Inc.), the plates were incubated with culture supernatants, followed by the addition of peroxidase‐conjugated anti‐mouse IgG diluted 1:2000 (Dako; Agilent Technologies, Inc.). The enzymatic reaction was conducted using a 1‐Step Ultra TMB‐ELISA (Thermo Fisher Scientific Inc.). Optical density was measured at 655 nm using an iMark microplate reader (Bio‐Rad Laboratories Inc.).

### Flow cytometry

Cell lines were harvested after brief exposure to 0.25% Trypsin/1 mmol/L EDTA (Nacalai Tesque, Inc.). After washing with 0.1% BSA in PBS, the cells were treated with primary mAbs for 30 min at 4°C, followed by treatment with Oregon Green 488‐conjugated to goat anti‐mouse IgG or anti‐rat IgG (Thermo Fisher Scientific Inc.). Fluorescence data were acquired using a Cell Analyzer EC800 (Sony Corp., Tokyo, Japan).

### Immunohistochemical analyses

Four‐*μ*m‐thick tissue sections were deparaffinized using xylene and rehydrated. After antigen retrieval, (autoclaving using citrate buffer, pH 6.0), sections were incubated with 1 *μ*g/mL of LpMab‐21 for 1 h at room temperature, and immunocomplexes were treated with an Envision+ Kit (Dako) for 30 min, color was developed using 3, 3‐diaminobenzidine tetrahydrochloride (DAB, Dako) for 5 min. Sections were then counterstained with hematoxylin (Wako Pure Chemical Industries Ltd.).

## Results

### Generation of a novel anti‐hPDPN mAb (LpMab‐21)

**Figure 1:**
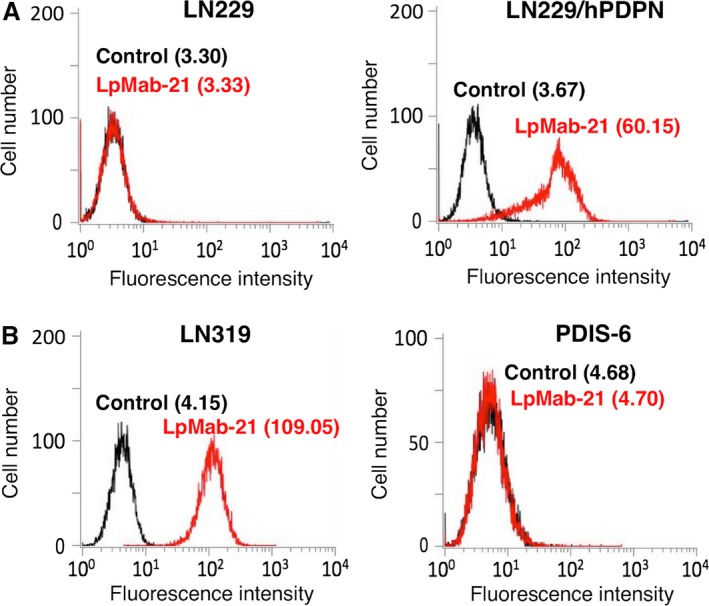
Flow cytometric analysis using LpMab‐21 to detect hPDPN expression. (A) LN229 and LN229/hPDPN cells were treated with LpMab‐21 (1 *μ*g/mL, red) or PBS (black) for 30 min at 4°C followed by treatment with anti‐mouse IgG‐Oregon green. (B) LN319 and LN319/hPDPN‐KO cells (PDIS‐6) were treated with LpMab‐21 (1 *μ*g/mL, red) or PBS (black) for 30 min at 4°C followed by addition of anti‐mouse IgG‐Oregon green. Fluorescence data were collected using a Cell Analyzer EC800. Geometric Mean was described. hPDPN, Human podoplanin.

We first immunized one mouse with the LN229/hPDPN, and harvested spleen cells were fused with P3U1. The ELISA screening was performed with supernatants from 960 hybridomas. Among 135 ELISA‐positive wells, 19 wells reacted with LN229/hPDPN, but not with LN229 in flow cytometry. We performed single‐cell cloning for 19 wells by limiting dilution, and could obtain 14 hybridomas. Among them, we previously reported five clones including LpMab‐10, LpMab‐12, LpMab‐13, LpMab‐17, and LpMab‐19 30, 31, 32, 33, 34, 35, 36, 38, 39, 43. In this study, we newly report LpMab‐21 (IgG_2a_, kappa), which was the first IgG_2a_ mouse anti‐hPDPN mAb in our study. Flow cytometry revealed that LpMab‐21 reacted with LN229/hPDPN cells but not with LN229 cells that were hPDPN‐negative (Fig. 1A). LpMab‐21 detected endogenous hPDPN, which is expressed in the glioblastoma cell line LN319, but not in LN319/hPDPN‐KO cells (PDIS ‐ 6), indicating that LpMab‐21 is specific against hPDPN (Fig. 1B).

**Figure 2:**
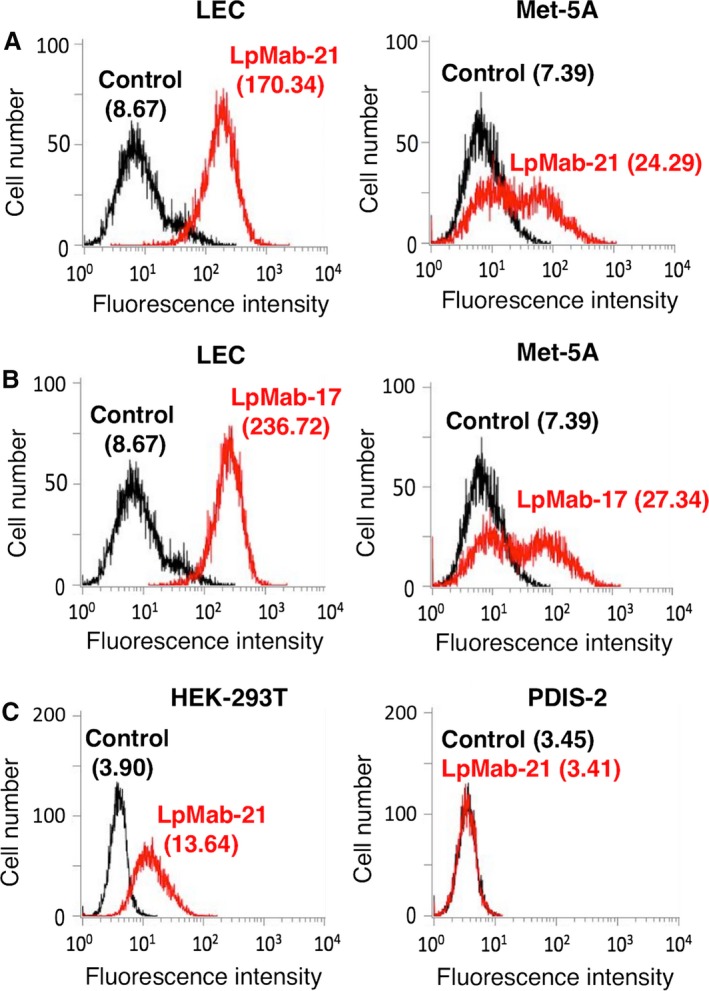
Flow cytometric analysis using LpMab‐21 to detect hPDPN expression in normal cells. (A) Human lymphatic endothelial cell (LEC) and human mesothelial cells (Met‐5A) were reacted with LpMab‐21 (1 *μ*g/mL, red) or PBS (black) for 30 min at 4°C, followed by treatment with anti‐mouse IgG‐Oregon green. (B) LEC and Met‐5A cells were treated with LpMab‐17 (1 *μ*g/mL, red) or PBS (black) for 30 min at 4°C, followed by treatment with anti‐mouse IgG‐Oregon green. (C) The human embryonic renal epithelial cell line (HEK‐293T) and HEK‐293T/hPDPN‐KO cells (PDIS‐2) were reacted with LpMab‐21 (1 *μ*g/mL, red) or PBS (black) for 30 min at 4°C, followed by addition of anti‐mouse IgG‐Oregon green. Fluorescence data were acquired using a Cell Analyzer EC800. Geometric Mean was described. LEC, lymphatic endothelial cell.

LpMab‐21 detected the expression of hPDPN in normal cells such as a lymphatic endothelial cell (LEC) and a mesothelial cell line Met‐5A (Fig. 2A). The positive‐control LpMab‐17 reacted with LEC and Met‐5A (Fig. 2B). LpMab‐21 detected endogenous hPDPN, which is expressed in the kidney epithelial cell line HEK‐293T, but not in HEK‐293T/hPDPN‐KO cells (PDIS‐2), indicating that LpMab‐21 is specific against hPDPN (Fig. 2C).

**Figure 3:**
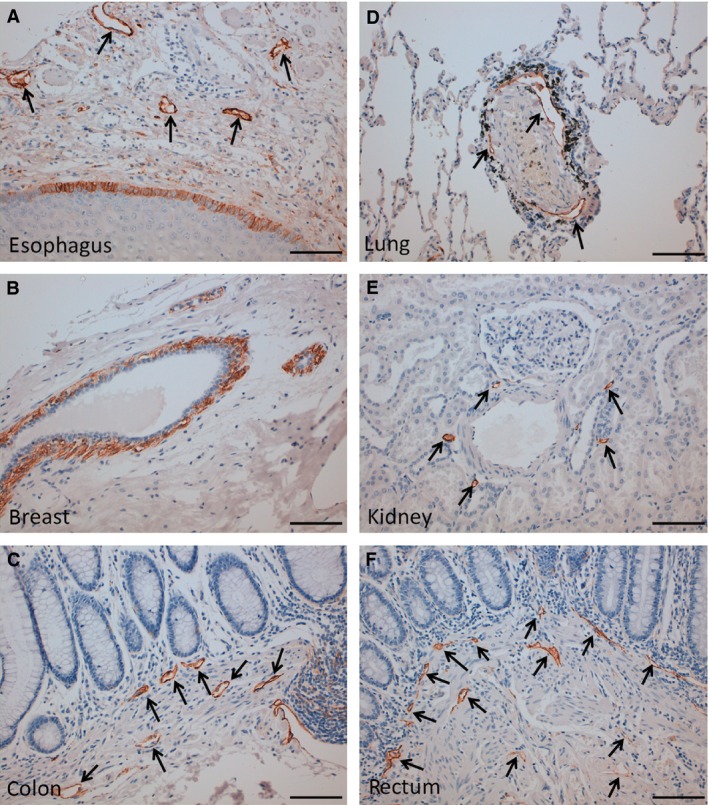
Immunohistochemical analysis using LpMab‐21 to detect PDPN expression in normal human tissues. Tissues harvested from the esophagus (A), breast (B), colon (C), lung (D), kidney (E), and rectum (F). After antigen retrieval procedure, sections were incubated with 1 *μ*g/mL of LpMab‐21, reacted with the Envision+ kit, color was developed, using DAB, and samples were then counterstained with hematoxylin. Arrowheads, lymphatic endothelial cells. Scale bar , 100 *μ*m. 3, DAB, 3‐diaminobenzidine tetrahydrochloride.

We next investigated whether LpMab‐21 was suitable for immunohistochemical analyses (Fig. 3). Consistent with the expression of hPDPN by lymphatic endothelial cells 44, LpMab‐21 reacted with lymphatic endothelial cells of the esophagus (Fig. 3A), colon (Fig. 3C), lung (Fig. 3D), kidney (Fig. 3E), and rectum (Fig. 3F). LpMab‐21 detected hPDPN expressed by basal keratinocytes of the esophagus (Fig. 3A) and myoepithelial cells of breast glands (Fig. 3B). In contrast, LpMab‐21 did not detect hPDPN expression in type I alveolar cells of lung (Fig. 3D) and podocytes of the renal glomerulus (Fig. 3E). These results indicate that the epitope recognized by LpMab‐21 is tissue‐specific 45.

### Flow cytometric and immunohistochemical analyses using LpMab‐21 to detect hPDPN expression in cancers

**Figure 4:**
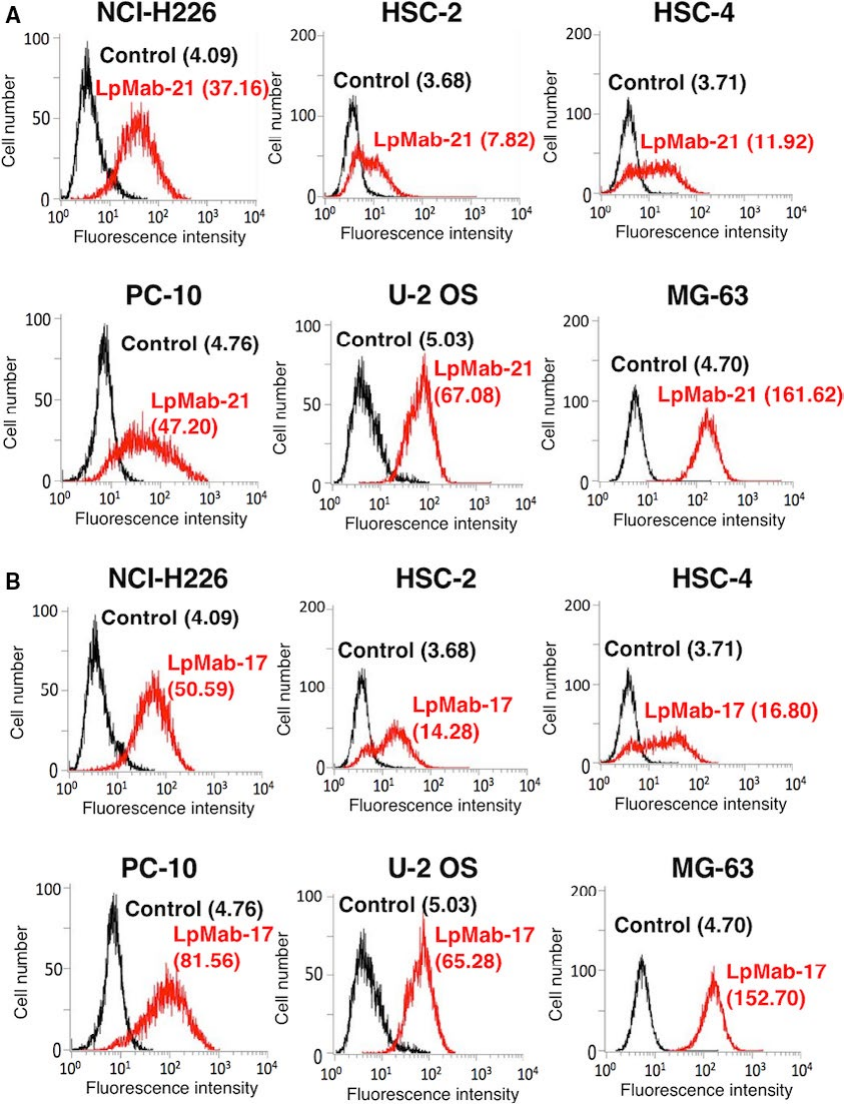
Flow cytometric analysis using LpMab‐21 to detect PDPN expression in human cancer cells. Human cell lines analyzed were as follows: mesothelioma, NCI‐H226; oral squamous cell carcinomas, HSC‐2 and HSC‐4; lung squamous cell carcinoma, PC‐10; and osteosarcomas U‐2 OS and MG‐63. Cells were treated reacted with LpMab‐21 (A) 1 *μ*g/mL; red), LpMab‐17 (B, 1 *μ*g/mL; red), or PBS (A and B, black) for 30 min at 4°C, followed by treatment with anti‐mouse IgG‐Oregon green. Fluorescence data were acquired using a Cell Analyzer EC800. Geometric Mean was described.

PDPN is expressed by cancers such as brain tumors, mesotheliomas, oral cancers, lung cancers, esophageal cancers, testicular cancers, and osteosarcoma 1, 9, 32. Flow cytometry using LpMab‐21 detected endogenous expression of PDPN by the human cancer cell lines as follows: mesothelioma, NCI‐H226; oral squamous cell carcinoma, HSC‐2 and HSC‐4; squamous cell carcinoma of the lung, PC‐10; and human osteosarcoma, U‐2 OS and MG‐63 (Fig. 4A). LpMab‐17 detected PDPN expression by all cell lines (Fig. 4B).

**Figure 5:**
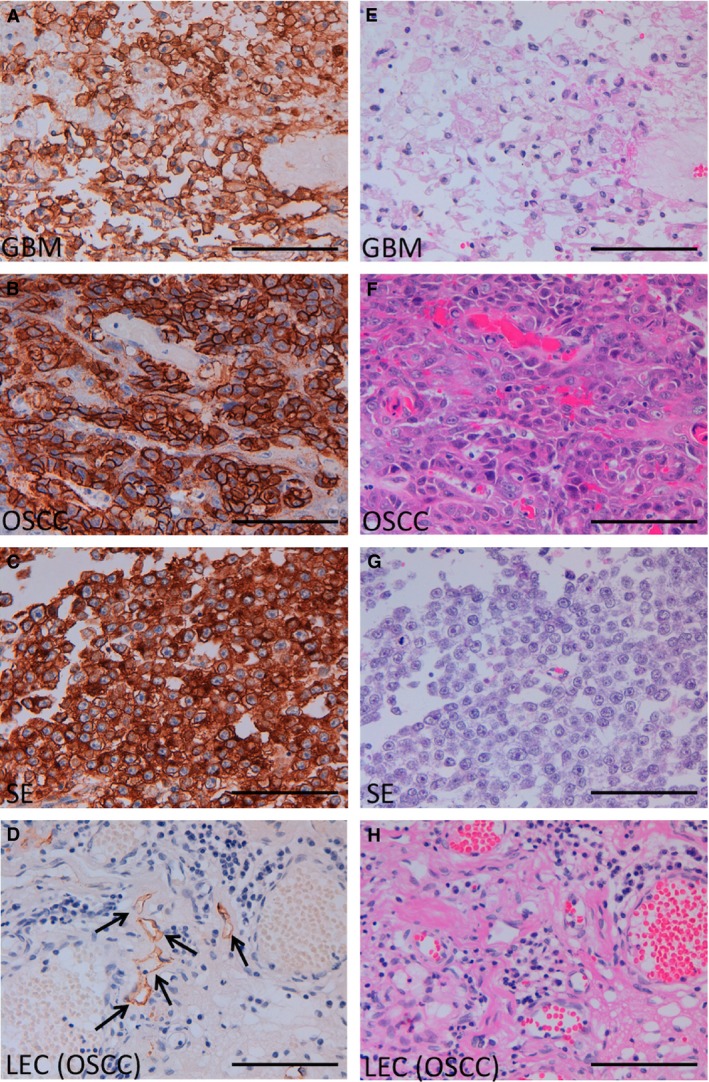
Immunohistochemical analysis using LpMab‐21 to detect PDPN expression in human cancer tissues. Tissue sections were prepared from the human cancer tissues as follows: glioblastoma (GBM, A and E); oral squamous cell carcinoma (OSCC; B, D, F, and H); seminoma (SE, C and G). Sections were incubated (antigen retrieval omitted) with 1 *μ*g/mL of LpMab‐21 (A–D), reacted with the Envision+ kit, color was developed using DAB, and samples were then counterstained with hematoxylin. Sections were stained with hematoxylin and eosin as well (E–H). Arrowheads: lymphatic endothelial cells. Scale bar = 100 *μ*m. DAB, 3‐diaminobenzidine tetrahydrochloride

Immunohistochemical analysis (with or without antigen retrieval), using LpMab‐21 detected membrane‐associated PDPN expression in the human tumor tissues as follows: glioblastoma (Fig. 5A), oral squamous cell carcinoma (OSCC) (Fig. 5B), and a seminoma (Fig. 5C). LpMab‐21 reacted with lymphatic endothelial cells in OSCC tissues (Fig. 5D), but not with vascular endothelial cells (Fig. 5D), demonstrating that LpMab‐21 is useful for detecting lymphatic endothelial cells in cancer tissues.

### Characterization of LpMab‐21 using glycan‐deficient cell lines

**Figure 6:**
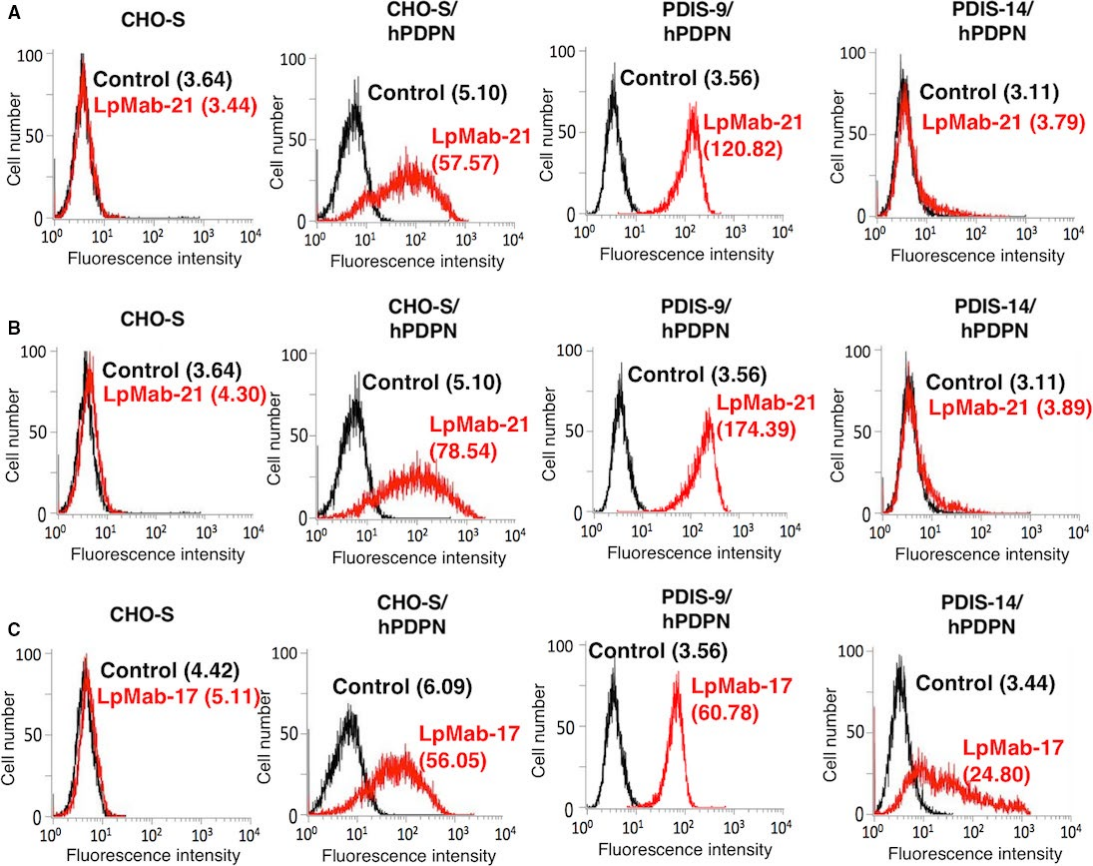
Flow cytometric analysis using LpMab‐21 to detect hPDPN expression in sialic acid‐deficient cells. CHO‐S, CHO‐S/hPDPN, PDIS‐9/hPDPN, and PDIS‐14/hPDPN cells were reacted with LpMab‐21 (A, 1 *μ*g/mL; red), LpMab‐21 (B, 10 *μ*g/mL; red), or LpMab‐17 (C, 1 *μ*g/mL; red), or PBS (A, B, and C; black) for 30 min at 4°C, followed by treatment with anti‐mouse IgG‐Oregon green. Fluorescence data were acquired using a Cell Analyzer EC800. Geometric Mean was described.

Human PDPN is *O*‐glycosylated, but not *N*‐glycosylated 6, 24, 46, 47, 48. In this study, we generated a GnT‐1‐knockout (KO) cell line (CHO‐S/GnT‐1‐KO, PDIS‐9) and a CMP‐sialic acid transporter (SLC35A1)‐KO cell line (CHO‐S/SLC35A1‐KO, PDIS‐14) by transfecting them with TALEN and CRISPR/Cas9 plasmids, respectively (Table 1). PDIS‐9 and PDIS‐14 cells were screened using leukoagglutinin from *Phaseolus vulgaris* (L‐PHA) and MAL‐II, respectively. When we used the hPDPN expression vector to transfect PDIS‐9 and PDIS‐14 cells, we found that LpMab‐21 reacted with CHO‐S/hPDPN and PDIS‐9/hPDPN cells but not with PDIS‐14/hPDPN cells (Fig. 6A and B). We further generated a GnT‐1‐KO cell line (HEK‐293T/GnT‐1‐KO, PDIS‐1 or PDIS‐12) and a CMP‐sialic acid transporter (SLC35A1)‐KO cell line (HEK‐293T/SLC35A1‐KO, PDIS‐22) by transfecting them with TALEN and CRISPR/Cas9 plasmids, respectively (Table 1). PDIS‐1 and PDIS‐12 cells were screened using L‐PHA. PDIS‐22 cells were screened using MAL‐II. We found that LpMab‐21 reacted with HEK‐293T, PDIS‐1, and PDIS‐12 cells but not with PDIS‐22 cells (Fig. S1). These results indicate that the hPDPN epitope recognized by LpMab‐21 includes a peptide sequence linked to sialic acid.

**Table 1:** Characterization of glycan‐deficient or PDPN‐knock out cells

Cell name	Parental cells	Targeted genes	Genom editing	Deficient
PDIS‐1	HEK‐293T	hsMgat1/GnT‐1	TALEN	*N*‐glycan
PDIS‐2	HEK‐293T	PDPN	CRISPR/Cas9	PDPN
PDIS‐6	LN319	PDPN	CRISPR/Cas9	PDPN
PDIS‐9	CHO‐S	hsMgat1/GnT‐1	TALEN	*N*‐glycan
PDIS‐12	HEK‐293T	hsMgat1/GnT‐1	TALEN	*N*‐glycan
PDIS‐14	CHO‐S	SLC35A1	CRISPR/Cas9	Sialic acid
PDIS‐22	HEK‐293T	SLC35A1	CRISPR/Cas9	Sialic acid

### Epitope mapping of LpMab‐21

**Figure 7:**
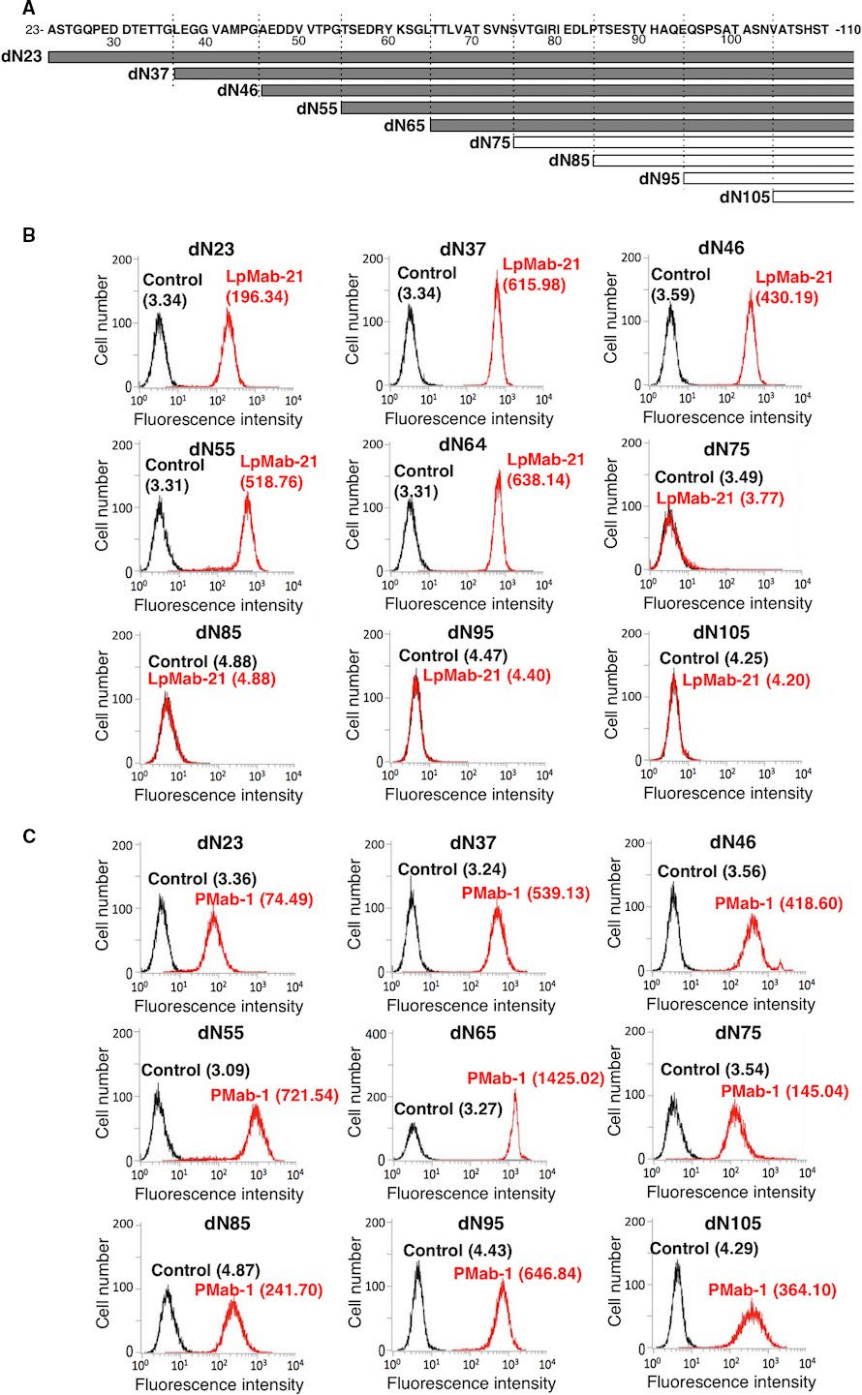
Epitope mapping of LpMab‐21 using deletion mutants of hPDPN. (A) Structures of hPDPN deletion dN23, dN37, dN46, dN55, dN64, dN75, dN85, dN95, dN105. (B, C) Each hPDPN deletion mutant was reacted with LpMab‐21 (B, 1 *μ*g/mL; red), PMab‐1 (C, 1 *μ*g/mL; red), or PBS (B and C, black) for 30 min at 4°C, followed by treatment with anti‐mouse IgG‐Oregon green (B) or anti‐rat IgG‐Oregon green (C). Fluorescence data were acquired using a Cell Analyzer EC800. Geometric Mean was described.

We expressed hPDPN deletion mutants in CHO‐K1 cells (Fig. 7A). LpMab‐21 detected dN23, dN37, dN46, dN55, and dN64. In contrast, LpMab‐21 did not react with dN75, dN85, dN95, or dN105, indicating that the N‐terminus of the epitope recognized by LpMab‐21 resides between hPDPN Thr65 and Val75 (Fig. 7B). All deletion mutants were detected, using the anti‐MAP‐tag mAb, PMab‐1 (Fig. 7C).

**Figure 8:**
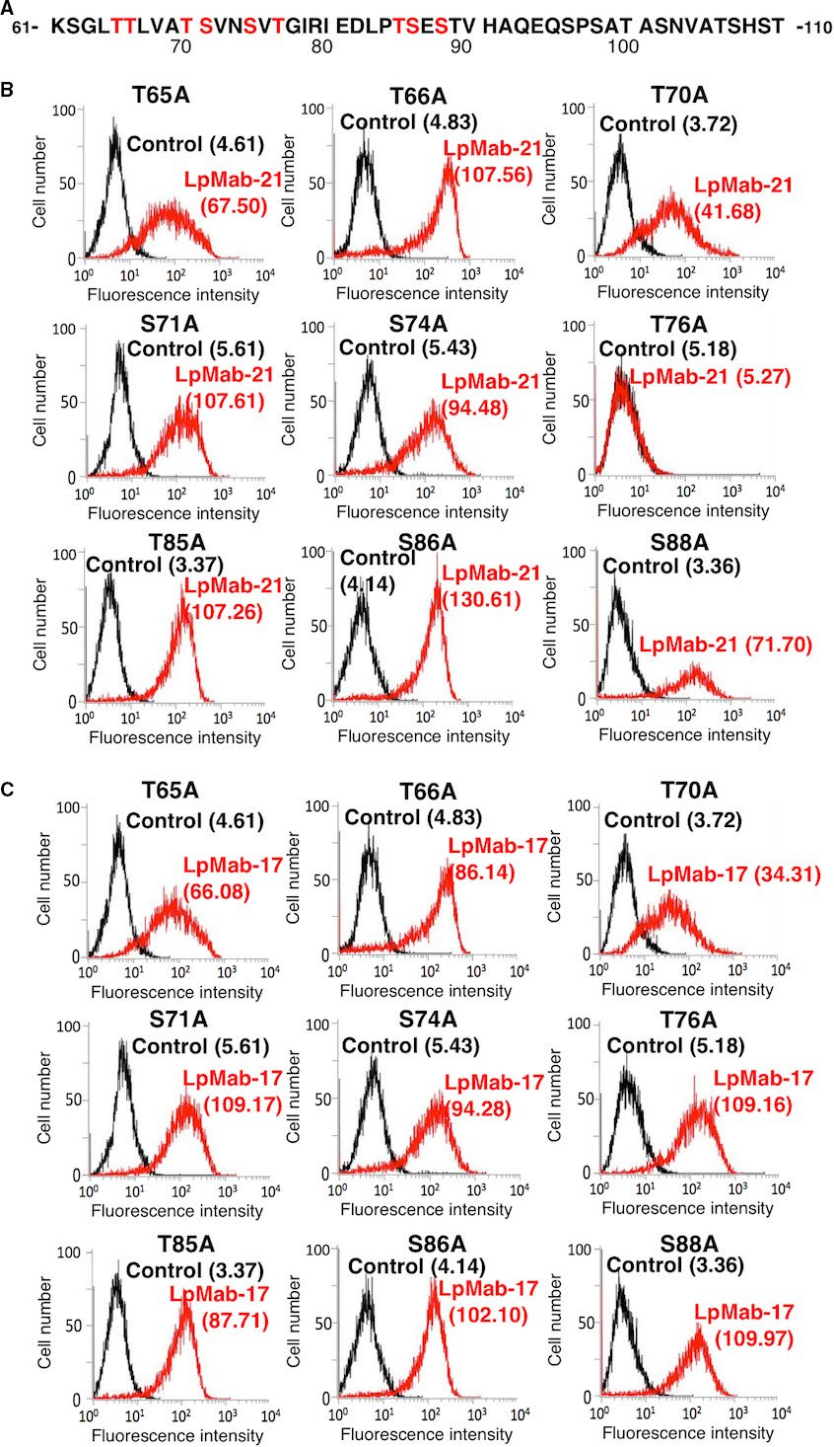
Epitope mapping of LpMab‐21 using hPDPN‐Ser/Thr point mutants. (A) Amino acid sequence of hPDPN encompassing Thr76. (B, C) Stable CHO‐S transfectants expressing hPDPN point mutants T65A, T66A, T70A, S71A, S74A, T76A, T85A, S86A, and S88A were reacted with LpMab‐21 (B, 1 *μ*g/mL; red), LpMab‐17 (C, 1 *μ*g/mL; red), or PBS (B and C; black) for 30 min at 4°C, followed by treatment with anti‐mouse IgG‐Oregon green. Fluorescence data were acquired using a Cell Analyzer EC800. Geometric Mean was described.

Next, we generated stable CHO‐S cell lines expressing the hPDPN point mutants T65A, T66A, T70A, S71A, S74A, T76A, T85A, S86A, and S88A (Fig. 8A). These mutants were chosen, because the epitope of LpMab‐21 includes a sialylated *O*‐glycan (Fig. 6) and starts between residues Thr65 and Val75 (Fig. 7). LpMab‐21 reacted with all transfectants, except for T76A (Fig. 8B). All point mutants targeting Ser/Thr residues were detected by LpMab‐17 (Fig. 8C). Together, these data support the conclusion that the epitope recognized by LpMab‐21 includes sialic acid linked to PDPN Thr76.

**Figure 9:**
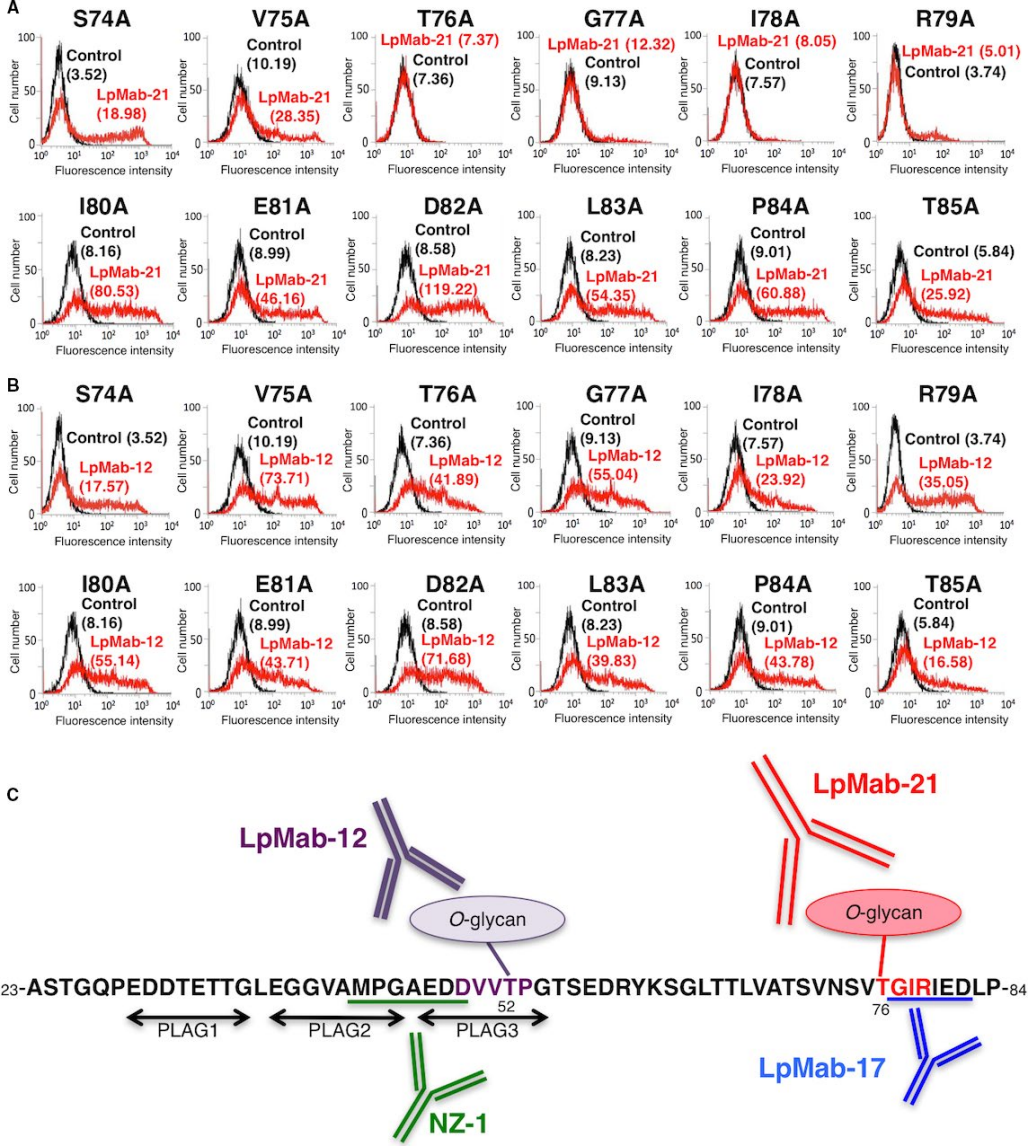
Epitope mapping of LpMab‐21, using point mutants of hPDPN. Nine hPDPN point Ser74–Thr85 point mutants were transiently expressed in CHO‐K1 cells. Cells were reacted with LpMab‐21 (A, 1 *μ*g/mL; red), LpMab‐12 (B, 1 *μ*g/mL; red), or control PBS (A and B, black) for 30 min at 4°C, followed by treatment with anti‐mouse IgG‐Oregon green. Fluorescence data were acquired using a Cell Analyzer EC800. (C) Illustration of the epitope recognized by anti‐hPDPN mAbs. Geometric Mean was described. PBS, phosphate‐buffered saline.

We used alanine scanning to localize the epitope recognized by LpMab‐21 near hPDPN Thr76. Thus, nine hPDPN point mutants (Ser74–Thr85) were transiently expressed in CHO‐K1 cells. LpMab‐21 did not detect T76A, G77A, I78A, or R79A. In contrast, LpMab‐12, which recognizes an epitope comprising hPDPN Thr52, detected each point mutant (Fig. 9B), indicating that Thr76–Arg79 is the minimum epitope recognized by LpMab‐21. We summarized and compared LpMab‐21 with the several anti‐hPDPN mAbs (Table 2).

**Table 2:** Characterization of anti‐PDPN glycopeptide mAbs

Anti‐glycopeptide mAb	Subclass	Epitope		CasMab/non‐CasMab	HC (LEC, T1a, POD)
		O‐glycan	Pepitde		
LpMab‐21	IgG2a, kappa	Thr76	Thr76–Arg79	Non‐CasMab	positive for only LEC
LpMab‐2	IgG1, kappa	Thr55/Ser56	Thr55–Leu64	CasMab	negative
LpMab‐3	IgG1, kappa	Thr76	Thr76–Glu81	Non‐CasMab	positive for LEC, T1a, POD
LpMab‐9	IgG1, kappa	Thr25	Thr25–Asp30	Non‐CasMab	not applicable
LpMab‐12	IgG1, kappa	Thr52	Asp49–Pro53	Non‐CasMab	positive for LEC, T1a, POD
LpMab‐19	IgG2b, kappa	Thr76	Thr76–Arg79	Non‐CasMab	positive for LEC, T1a, POD

CasMab, cancer‐specific mAb; IHC, immunohistochemical analysis; T1a, type I alveolar cells; POD, podocyte.

## Discussion

The anti‐hPDPN mAb (NZ‐1) detects hPDPN with high specificity and sensitivity 6, 10, 25. Moreover NZ‐1, which is also useful for detecting the podoplanin/aggrus (PA) epitope tag 49, 50, is efficiently internalized by glioma cell lines, accumulates in tumors in vivo*,* and is therefore a suitable candidate for therapy for malignant gliomas 5, 10. Further, NZ‐1 inhibits tumor cell‐induced platelet aggregation and tumor metastasis 23. NZ‐1 mediates antibody‐dependent cellular cytotoxicity (ADCC) and complement‐dependent cytotoxicity (CDC) against tumor cells that express hPDPN 51. Moreover, NZ‐1 is suitable for western blotting, flow cytometry, immunohistochemistry, and immunoprecipitation 51. However, NZ‐1 was produced using synthetic peptide 6; therefore, further mAbs against hPDPN, especially anti‐glycopeptide mAbs are necessary to investigate the structure and function of PDPN.

Previously, we developed the original technology to produce cancer‐specific mAbs that detect cell type‐specific posttranslational modifications of the same protein 30. We used LN229/hPDPN cells as the immunogen to elicit novel anti‐PDPN mAbs. We produced several clones including LpMab‐2, LpMab‐3, and LpMab‐9 as anti‐glycopeptide mAbs 30. Recently, we further immunized mice with LN229/hPDPN cells to develop further anti‐glycopeptide mAbs against human PDPN, and characterized several clones, including LpMab‐12 and LpMab‐19. In this study, we characterized another clone LpMab‐21, which detects many human cancer cell lines that express PDPN, such as those derived from glioblastomas, lung squamous cell carcinomas, oral squamous cell carcinomas, osteosarcomas, and malignant mesotheliomas. The isotypes of previously established anti‐PDPN mAbs are IgG_1_ (seven clones) and IgG_3_ (one clone). However, the applications of mouse IgG_3_ mAbs are limited because they often aggregate 52. Moreover, mouse IgG_1_ and IgG_3_ isotypes do not induce ADCC or CDC. Therefore, we required chimeric mAbs using human IgG_1_ to investigate these activities 33. LpMab‐21 (IgG_2a_ subclass) could be used to investigate the function of anti‐tumor activities in xenograft models because LpMab‐21 induced ADCC and CDC (data not shown).

Furthermore, we need several glycan‐deficient cell lines such as sialic acid‐deficient or *N*‐glycan deficient cell lines to characterize those mAbs. In this study, we successfully produced several glycan‐deficient cell lines such as sialic acid deficient (PDIS‐14 and PDIS‐22) or *N*‐glycan deficient cell lines (PDIS‐1, PDIS‐9, and PDIS‐12), using CRISPR/Cas9 and TALEN systems. Using those cell lines, we determined that the epitope of LpMab‐21 includes sialic acids, indicating that we can also investigate whether the epitope of novel mAbs against the other membrane proteins possesses sialic acids or *N*‐glycans.

We showed here that LpMab‐21 detected glioblastomas, oral cancers, and seminomas (Fig. 5) as well as normal cells such as lymphatic endothelial cells, basal epithelial cells of the esophagus, and myoepithelial cells of breast glands (Fig. 3). In contrast, LpMab‐21 did not react with the renal glomerulus or with type I alveolar cells of lung (Fig. 3), indicating that sialylation of hPDPN is tissue‐specific.

In conclusion, LpMab‐21 shows promise for investigating the expression and function of hPDPN in cancers and normal tissues. Further, mAbs that recognize different epitopes of hPDPN should serve as powerful tools for identifying the function of hPDPN.

## Conflict of Interest

No declared.

## Supporting information


**Figure S1.** Flow cytometric analysis, using LpMab‐21 to detect hPDPN expression in sialic acid‐deficient cells. HEK‐293T, PDIS‐1, PDIS‐12, and PDIS‐22 cells were reacted with LpMab‐21 (A, 1 *μ*g/mL; red), or LpMab‐17 (B, 1 *μ*g/mL; red), or PBS (A and B; black) for 30 min at 4°C, followed by treatment with anti‐mouse IgG‐Oregon green. Fluorescence data were acquired using a Cell Analyzer EC800.
