# Supplementary figures and images for: Development and characterization of anti‐glycopeptide monoclonal antibodies against human podoplanin, using glycan‐deficient cell lines generated by CRISPR/Cas9 and TALEN
Source: Cancer Med. 2017 Jan 19;6(2):382–96. doi: 10.1002/cam4.954 (PMC5313638; doi:10.1002/cam4.954)

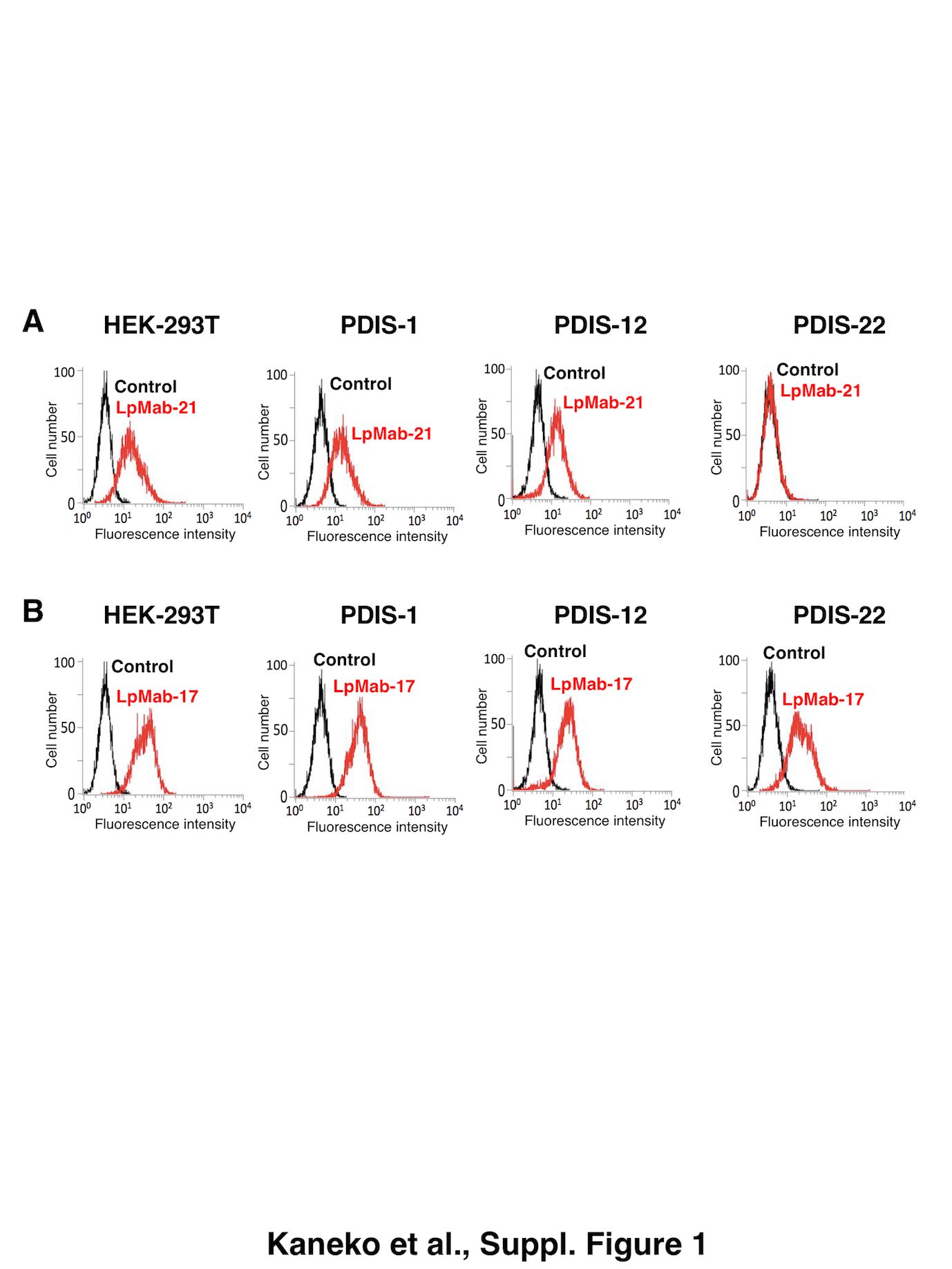

Supplement: Supplementary file 1 — Figure S1. Flow cytometric analysis, using LpMab‐21 to detect hPDPN expression in sialic acid‐deficient cells. HEK‐293T, PDIS‐1, PDIS‐12, and PDIS‐22 cells were reacted with LpMab‐21 (A, 1 μg/mL; red), or LpMab‐17 (B, 1 μg/mL; red), or PBS (A and B; black) for 30 min at 4°C, followed by treatment with anti‐mouse IgG‐Oregon green. Fluorescence data were acquired using a Cell Analyzer EC800. [file CAM4-6-382-s001.tiff]
