# Supplementary material for: Development and characterization of anti‐glycopeptide monoclonal antibodies against human podoplanin, using glycan‐deficient cell lines generated by CRISPR/Cas9 and TALEN
Source: Cancer Med. 2017 Jan 19;6(2):382–96. doi: 10.1002/cam4.954 (PMC5313638; doi:10.1002/cam4.954)
Supplement: Supplementary file 2 [file CAM4-6-382-s002.docx]

**Supplementary information**

**Supplementary Figure 1. Flow cytometric analysis using LpMab-21 to detect hPDPN expression in sialic acid-deficient cells.** HEK-293T, PDIS-1, PDIS-12, and PDIS-22 cells were reacted with LpMab-21 (A, 1 μg/ml; red), or LpMab-17 (B, 1 μg/ml; red), or PBS (A and B; black) for 30 min at 4°C, followed by treatment with anti-mouse IgG-Oregon green. Fluorescence data were acquired using a Cell Analyzer EC800.
